# Cranial Ultrasound Morphometry and Head Circumference at Term-Equivalent Age Are Associated with Early Neurodevelopmental Outcomes in Preterm Infants

**DOI:** 10.3390/medicina62071393

**Published:** 2026-07-18

**Authors:** Dorotea Drašković, Maja Zaninović, Koraljka Manestar Rukavina, Maja Ješić, Ana Milardović, Iva Bilić Čače

**Affiliations:** 1 Department of Pediatrics, Clinical Hospital Centre Rijeka, 51000 Rijeka, Croatia; maja.zaninovic@medri.uniri.hr (M.Z.); koraljkamr@medri.uniri.hr (K.M.R.); maja.jesic@medri.uniri.hr (M.J.); ana.milardovic@medri.uniri.hr (A.M.); iva.bilic@medri.uniri.hr (I.B.Č.); 2 Faculty of Medicine, University of Rijeka, 51000 Rijeka, Croatia

**Keywords:** cerebral ventricles, child development, infant, premature, ultrasonography

## Abstract

*Background and Objectives:* Advances in perinatal and neonatal care have improved survival in preterm infants, yet neurodevelopmental impairment remains a major concern. A considerable proportion of preterm infants with normal or only mildly abnormal neuroimaging findings still develop neurodevelopmental difficulties, highlighting the need for more sensitive imaging markers beyond the detection of major brain lesions. Cranial ultrasound is a widely used bedside imaging tool that enables both detection of brain injury and quantitative assessment of brain growth. This study aimed to evaluate the association between early and term-equivalent cranial ultrasound morphometric parameters, head circumference, and neurodevelopmental outcomes assessed by the Bayley Scales of Infant and Toddler Development, Fourth Edition (Bayley-4). *Materials and Methods:* This exploratory observational study included 22 preterm infants admitted to a neonatal intensive care unit. Cranial ultrasound examinations were performed within the first 72 h of life and at term-equivalent age (37–40 weeks postmenstrual age). Measured parameters included the Levene index, lateral ventricular width, third ventricle width, subarachnoid space, and interhemispheric fissure. Head circumference was analyzed as an additional marker of postnatal brain growth. Neurodevelopment was assessed at 12–14 months corrected age using Bayley-4. Associations were analyzed using Spearman’s rank correlation. *Results*: Significant correlations were more frequent at term-equivalent age than in the early neonatal period. Early measurements showed positive associations between ventricular parameters and motor outcomes, as well as between head circumference and receptive communication (*p* < 0.01). Third ventricle width was also associated with receptive communication. At term-equivalent age, lateral ventricular width demonstrated consistent negative correlations with motor and language outcomes, with strong associations in motor domains (*p* < 0.01). Head circumference showed limited positive associations, reaching significance only for personal–social skills, while third ventricle width was associated with play and leisure activities. *Conclusions*: Morphometric cranial ultrasound measurements, particularly those obtained at term-equivalent age, may provide valuable additional information about early neurodevelopment in preterm infants. Head circumference reflected postnatal brain growth but showed less consistent associations with neurodevelopmental outcomes than ventricular morphometric parameters. Larger prospective studies with longer follow-up are needed to confirm these findings and define the prognostic value of individual cranial ultrasound morphometric parameters and head circumference for predicting neurodevelopmental outcomes in preterm infants.

## 1. Introduction

Advances in perinatal and neonatal care over recent decades have substantially improved the survival of preterm infants, particularly those born extremely preterm. However, improved survival has been accompanied by an increasing number of children who experience neurodevelopmental impairments, one of the most important long-term consequences of prematurity. It is estimated that approximately 20–40% of preterm infants develop some form of neurodevelopmental difficulty affecting motor, cognitive, language, or socioemotional domains. These difficulties may manifest as cerebral palsy, developmental coordination disorder, language delay, cognitive impairment, learning difficulties, attention-deficit/hyperactivity disorder, and behavioural problems.

Even in the absence of major structural brain injury, subtle developmental difficulties may occur later in childhood, in attention, executive functioning, and learning abilities [1,2]. The period between the 24th and 40th weeks of gestation is characterized by rapid maturation of oligodendrocytes and intensive white matter development, representing a phase of increased vulnerability to hypoxic–ischemic events, inflammation, hemodynamic instability, and metabolic disturbances. Pre-oligodendrocytes, which predominate during this period, are sensitive to oxidative stress and inflammation, accounting for the high incidence of white matter injury in preterm infants and its association with later motor and cognitive impairments [3,4]. White matter injury, intraventricular hemorrhage, and disturbances of brain maturation represent the most common neuropathological substrates of neurodevelopmental impairment in preterm infants. However, it is well recognized that a considerable proportion of infants with normal or only mildly abnormal neuroimaging findings may still experience functioning difficulties. This suggests that an approach focused exclusively on detecting major lesions is insufficient for precise assessment of neurodevelopmental risk [5].

Cranial ultrasound is the primary imaging modality for evaluating the brain in preterm infants due to its availability, safety, bedside applicability, and suitability for serial examinations. Ultrasound enables the detection of intraventricular hemorrhage, post-hemorrhagic ventricular dilatation, periventricular leukomalacia, and other pathological changes, but also allows quantitative measurements of brain structures and cerebrospinal fluid spaces [6]. Although numerous cranial ultrasound morphometric measurements have been described, parameters such as the Levene index, lateral ventricular width, interhemispheric fissure, and subarachnoid space remain insufficiently explored as predictors of neurodevelopment. These measurements are simple and easy to perform in everyday neonatal practice, making them particularly suitable for widespread clinical application. These parameters may reflect the relationship between brain parenchymal growth and cerebrospinal fluid spaces and may indirectly indicate disturbances in brain maturation [6]. Unlike qualitative assessment of lesions, morphometric measurements enable the continuous monitoring of development and potentially earlier identification of abnormalities, even in the absence of clearly visible pathological changes [6]. Assessment of the brain at term-equivalent age is important because findings at this stage reflect the cumulative effects of prenatal and postnatal factors, including respiratory complications, infections, hemodynamic disturbances, and nutritional status. Previous studies have shown that ultrasound findings at term-equivalent age may have greater prognostic value for later neurodevelopment than findings obtained in the early neonatal period, largely because they allow for the evaluation of brain growth dynamics and maturation of brain parenchyma [7]. Standardized developmental tests are widely used for objective assessment of neurodevelopmental in early childhood. Among these, the Bayley Scales of Infant and Toddler Development are considered an international standard. The most recent version, Bayley-4, enables detailed assessment of cognitive, motor, language, socioemotional development, and adaptive behaviour and has improved psychometric properties compared with earlier versions of the test [8]. Despite their widespread use, methodological limitations and challenges in interpreting Bayley results are frequently discussed in the literature, particularly in preterm populations. Some studies suggest that developmental impairments may be underestimated in early assessments, emphasizing the importance of long-term follow-up in this population [9]. There is a growing interest in early biomarkers of neurodevelopmental outcomes. However, most previous studies have focused on severe ultrasound lesions or on single measurements at limited time points, without systematic evaluation of dynamic changes. Furthermore, relatively few studies have analyzed morphometric parameters as continuous variables and examined their associations with specific developmental domains, such as cognitive, language, and motor outcomes. A better understanding of these relationships could contribute to the development of more reliable early risk-assessment models and enable a more individualized approach to follow-up and early intervention in preterm infants.

The aim of this study was to evaluate the associations between early and term-equivalent cranial ultrasound morphometric parameters, head circumference, and neurodevelopmental outcomes assessed using the Bayley Scales of Infant and Toddler Development, Fourth Edition (Bayley-4), hypothesizing that these parameters would be associated with early neurodevelopmental outcomes in preterm infants.

## 2. Materials and Methods

### 2.1. Study Design and Setting

This exploratory study included preterm infants born before 36 weeks of gestation and admitted to the neonatal intensive care unit at the Clinical Hospital Centre Rijeka, Croatia. According to local clinical protocols, all infants born before 36 weeks of gestation are routinely admitted to the neonatal intensive care unit or a high-dependency neonatal care unit for monitoring and management.

All infants underwent cranial ultrasound examinations both in the early neonatal period (within the first 72 h of life) and at term-equivalent age (37–40 weeks of corrected gestational age) and were subsequently enrolled in a standardized neurodevelopmental follow-up program. Neurodevelopmental outcomes were assessed at 12–14 months of corrected age using the Bayley Scales of Infant and Toddler Development, Fourth Edition (Bayley-4).

### 2.2. Participants

#### 2.2.1. Inclusion Criteria

• Gestational age at birth < 36 weeks;
• Written informed parental consent for participation in the study;
• Available cranial ultrasound measurements within the first 72 h of life;
• Available cranial ultrasound measurements at term-equivalent age (37–40 weeks postmenstrual age);
• Available Bayley-4 neurodevelopmental assessment at 12–14 months corrected age.

#### 2.2.2. Exclusion Criteria

• Incomplete ultrasound measurements at any of the predefined time points;
• Unavailable or incomplete neurodevelopmental assessment;
• Major congenital malformations of the central nervous system;
• Genetic syndromes known to affect neurodevelopment independently.

All included infants were treated according to standard clinical protocols of the neonatal intensive care unit.

### 2.3. Cranial Ultrasound Examination

Cranial ultrasound examinations were performed using a standard technique through the anterior fontanelle by one of three experienced pediatricians trained in neonatal neurosonography, using a Mindray DC series ultrasound system with a microconvex transducer (5–8 MHz). Before the start of the study, the examiners agreed on standardized anatomical landmarks and measurement planes according to established neonatal cranial ultrasonography guidelines. Examinations were performed within the first 72 h of life and at term-equivalent age (37–40 weeks postmenstrual age), and all images were stored in DICOM format. The stored ultrasound images were subsequently reviewed independently by all three examiners, who were blinded to each other’s measurements. For each parameter, the mean of the three independent measurements was used for statistical analysis. Measurements were obtained in standard bilateral coronal planes at the level of the insula.

The following morphometric parameters were analyzed:

• Levene index (left and right);
• Lateral ventricular width (left and right);
• Subarachnoid space width;
• Interhemispheric fissure width;
• Third ventricle width.

All measurements were recorded in millimetres (mm). Decimal values were rounded to the nearest whole millimetre according to standard rounding rules (≤0.4 mm rounded down and ≥0.5 mm rounded up). Measurements were performed by three trained pediatricians experienced in neonatal neurosonography, using the same measurement protocol and anatomical landmarks to minimize interobserver variability. All measurements were digitally stored on the ultrasound system, and data were analyzed anonymously.

Head circumference at birth and the term-equivalent age (in centimetres (cm), rounded to 0.5 cm) were recorded.

### 2.4. Neurodevelopmental Assessment

Neurodevelopmental outcomes were assessed using the Bayley Scales of Infant and Toddler Development, Fourth Edition (Bayley-4). Testing was performed at 12–14 months of corrected age as part of the routine neurodevelopment follow-up program for preterm infants. Assessments were conducted by a trained pediatrician certified in Bayley-4 administration.

Standard scores were analyzed for the following domains:

• Cognitive development;
• Motor development (gross and fine motor skills);
• Language development (receptive communication, reflecting language comprehension, and expressive communication);
• Socioemotional development;
• Adaptive behaviour.

### 2.5. Variables and Data Collection

The following variables were collected:

• Gestational age at birth;
• Head circumference at birth and at term-equivalent age;
• Cranial ultrasound measurements in the early neonatal period (first 72 h of life);
• Cranial ultrasound measurements at term-equivalent age (37–40 weeks postmenstrual age);
• Bayley-4 results at 12–14 months corrected age.

### 2.6. Statistical Analysis

Statistical analysis was performed with the aim of preliminary variable screening and identification of potential associations between morphometric cranial ultrasound parameters and neurodevelopmental outcomes. All analyses were conducted using the R statistical environment (R Core Team, 2022) [10].

Descriptive statistics were presented using

• Medians;
• Interquartile ranges (Q1–Q3);
• Minimum and maximum values.

Associations between continuous variables were assessed using Spearman’s rank correlation coefficient. Given the exploratory nature of the study and the relatively small sample size, a non-parametric correlation method was considered the most appropriate approach for evaluating associations between continuous variables without assuming normal data distribution. Statistical significance was set at *p* < 0.05, and results with *p* < 0.01 were interpreted as highly statistically significant. The primary aim of the analysis was to identify potentially relevant associations between cranial ultrasound measurements and neurodevelopmental outcomes that could be further investigated and confirmed in future larger prospective studies.

### 2.7. Ethical Considerations

The study was conducted in accordance with the ethical principles of the Declaration of Helsinki. Data were collected and analyzed anonymously in accordance with personal data protection regulations. Parents or legal guardians provided informed consent for diagnostic and neurodevelopment procedures performed as part of standard clinical care, as well as for the use of anonymized data for scientific purposes. The study protocol was approved by the Ethics Committee of the Clinical Hospital Centre Rijeka.

## 3. Results

### 3.1. Participant Characteristics

Initially, 28 preterm infants were enrolled in the study. Six infants were excluded from the final analysis: five died before completion of follow-up (including two with severe congenital anomalies), and one infant was excluded because of a major neurological malformation (congenital hydrocephalus). The final study cohort consisted of 22 preterm infants, of whom 9 (40.9%) were male and 13 (59.1%) female. The mean gestational age at birth was 31.1 ± 2.8 weeks. The mean birth weight was 1540 ± 620 g, the mean birth length was 40 ± 5 cm, and the mean head circumference was 28.8 ± 3.0 cm. Major neonatal morbidities included culture-proven sepsis, bronchopulmonary dysplasia, retinopathy of prematurity, necrotizing enterocolitis, and intraventricular hemorrhage. Additional demographic, anthropometric, and clinical characteristics are presented in Table 1.

### 3.2. Descriptive Statistics of Neurodevelopmental Outcomes

**Figure 1:**
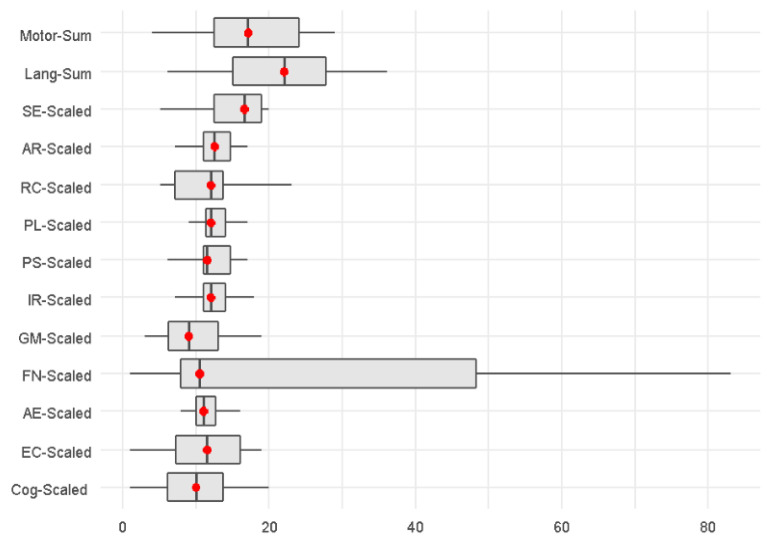
Distribution of Bayley-4 domain scores at 12–14 months corrected age.

Median standard scores across Bayley-4 domains ranged between 9 and 17 points. The greatest variability was observed in the motor domains, particularly in fine motor skills and total motor scores, whereas cognitive and most language domains showed narrower ranges of values. The socioemotional domain and adaptive behavior also demonstrated wider ranges of scores compared with most other domains. The distribution of results across individual Bayley domains is presented graphically using a boxplot diagram (Figure 1), illustrating medians, interquartile ranges, and total ranges. The greatest dispersion was observed in the fine motor domain, which showed the widest overall range of values. Increased variability was also observed in gross motor skills and total motor scores. Cognitive and language subdomains demonstrated more uniform distributions with narrower interquartile ranges. The socioemotional domain and composite adaptive behaviour scores showed wider distributions, and isolated outliers were observed in some domains.

### 3.3. Descriptive Statistics of Ultrasound Measurements

**Figure 2:**
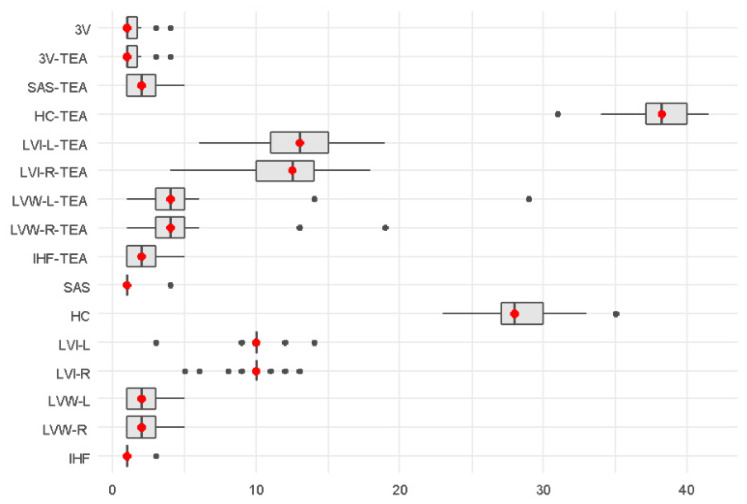
Distribution of morphometric cranial ultrasound parameters in the early neonatal period and at term-equivalent age.

**Figure 3:**
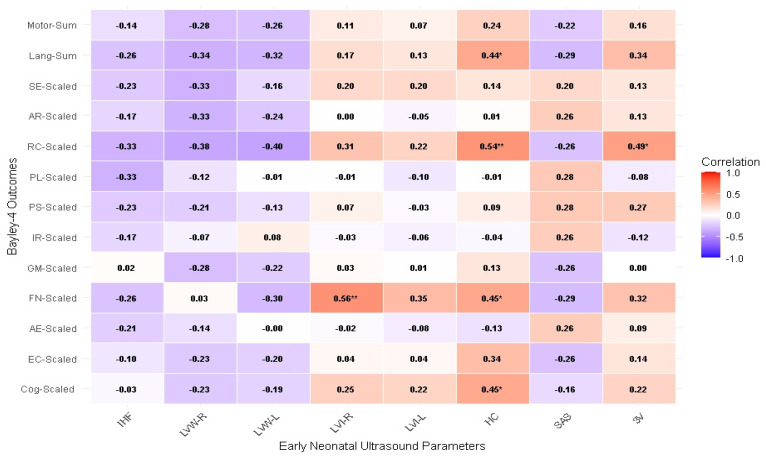
Spearman correlations between early neonatal ultrasound parameters and Bayley-4 outcomes. * Asterisks indicate statistical significance (* *p* < 0.05; ** *p* < 0.01).

Descriptive statistics of morphometric cranial ultrasound parameters and head circumference are presented in Figure 2. In the early neonatal period, values for the Levene index, lateral ventricular width, third ventricular width, subarachnoid space, and interhemispheric fissure showed relatively narrow ranges and low variability. At term-equivalent age, median values of most measured parameters were higher in comparison to the early neonatal period, with somewhat wider ranges, particularly in lateral ventricular measurements. Measurements of the third ventricle, subarachnoid space, and interhemispheric fissure remained relatively stable and showed low variability. Head circumference showed the expected increase in median values and overall range between the early neonatal period and term-equivalent age. The distribution of ultrasound variables in the early neonatal period and at term-equivalent age is illustrated in Figure 3.

### 3.4. Associations Between Ultrasound Parameters and Neurodevelopmental Outcomes

**Figure 4:**
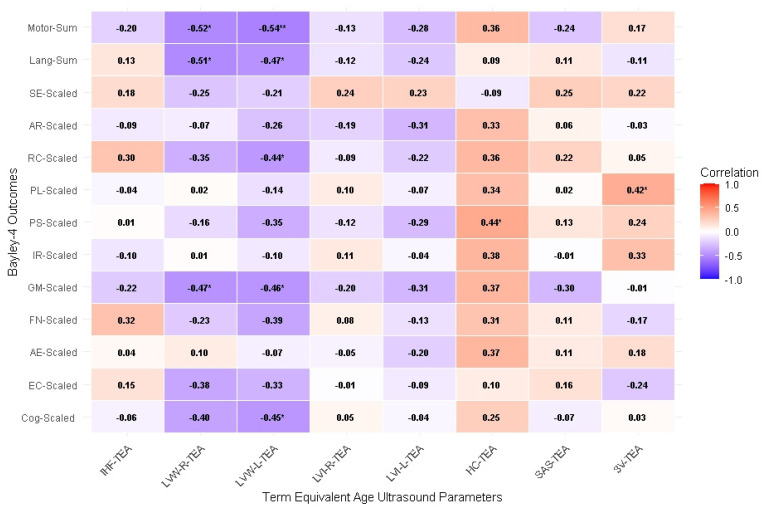
Spearman correlations between term-equivalent age ultrasound parameters and Bayley-4 outcomes. * Asterisks indicate statistical significance (* *p* < 0.05; ** *p* < 0.01).

In the early neonatal period, several moderate correlations were observed, primarily involving ventricular parameters and motor outcomes. A positive, statistically significant correlation was observed between ventricular measurements and fine motor performance, with correlation coefficients ρ = 0.56 (*p* < 0.01). In addition, head circumference demonstrated several positive correlations with developmental outcomes. A moderate positive correlation was observed with fine motor performance (ρ = 0.25) and language score (ρ = 0.44). The strongest association was observed between head circumference and receptive communication, with a statistically significant correlation (ρ = 0.54; *p* < 0.01). Furthermore, third ventricle width in the early neonatal period showed a statistically significant positive correlation with receptive communication (ρ = 0.49; *p* < 0.05). However, despite these individual associations, findings in the early neonatal period were not consistent across developmental domains. Most ultrasound parameters demonstrated weak or non-significant correlations with cognitive, language, and socioemotional outcomes, and no clear or systematic pattern of association was observed. The results are presented in Figure 3 and Figure 4.

At term-equivalent age, correlations were stronger and more consistent compared to those observed in the early neonatal period. Head circumference at term-equivalent age showed predominantly positive associations with neurodevelopmental outcomes, with a statistically significant moderate positive correlation observed for personal skills (ρ = 0.44; *p* < 0.05), while moderate positive but non-significant correlations were found for gross motor performance (ρ = 0.37), total motor score (ρ = 0.36), receptive communication (ρ = 0.36), and interpersonal relationships (ρ = 0.38), and a weak non-significant correlation for cognitive development (ρ = 0.25). Lateral ventricular width at term-equivalent age demonstrated the most consistent associations with neurodevelopmental outcomes, showing negative correlations across multiple domains. On the right side, statistically significant negative correlations were observed with total motor score (ρ = −0.52; *p* < 0.05), language score (ρ = −0.51; *p* < 0.05), and gross motor performance (ρ = −0.47; *p* < 0.05). On the left side of brain ventricular width showed statistically significant negative correlations with total motor score (ρ = −0.54; *p* < 0.01), language score (ρ = −0.47; *p* < 0.05), gross motor performance (ρ = −0.46; *p* < 0.05), receptive communication (ρ = −0.44; *p* < 0.05), and cognitive development (ρ = −0.45; *p* < 0.05). A statistically significant positive correlation was observed between third ventricle width at term-equivalent age and the domain of play and leisure activities (ρ = 0.42; *p* < 0.05). No other consistent or statistically significant associations were identified for this parameter. Other ultrasound parameters, including subarachnoid space, interhemispheric fissure, and Levene index, did not demonstrate consistent or statistically significant correlations with neurodevelopmental outcomes.

## 4. Discussion

This study analyzed the association between morphometric cranial ultrasound parameters, obtained in the early neonatal period and at term-equivalent age, head circumference as an anthropometric marker of postnatal brain growth, and neurodevelopmental outcomes assessed using the Bayley-4 scales in preterm infants.

The main finding of this study was that statistically significant associations with neurodevelopmental outcomes were more frequent and more consistent for measurements obtained at term-equivalent age than for those obtained in the early neonatal period. This finding is in line with the general concept that imaging performed closer to term better reflects cumulative brain growth and maturation in preterm infants than measurements obtained in the first days of life. Previous studies have similarly emphasized the prognostic importance of neuroimaging at term-equivalent age, particularly in relation to later neurodevelopmental outcomes [7]. While our findings are consistent with previous evidence, our study adds to the existing literature by examining the associations between individual cranial ultrasound morphometric parameters and specific Bayley-4 developmental domains.

In the early neonatal period, several significant associations were identified, including positive correlations between ventricular parameters and fine motor outcomes, between head circumference and receptive communication, and between third ventricle width and receptive communication. Because of the small sample size and exploratory design, these early associations should be interpreted with caution. Measurements obtained within the first 72 h of life primarily reflect the initial structural condition of the brain rather than the subsequent postnatal course, during which disturbances of brain maturation may evolve despite the absence of major structural brain injury [12]. For this reason, isolated early correlations may be less stable and less reflective of underlying brain development than associations observed at term-equivalent age [13,14,15].

At term-equivalent age, lateral ventricular width showed consistent associations across multiple neurodevelopmental domains. Larger ventricular widths on both sides were associated with less favorable motor and language outcomes, while on the left side additional associations were observed with cognitive and receptive communication scores. These findings suggest that increased ventricular size at term-equivalent age may reflect altered brain growth or relatively reduced surrounding brain parenchyma, representing a marker of vulnerability associated with adverse motor, language, and cognitive outcomes. Such an interpretation is biologically plausible, since ventricular enlargement in preterm infants is often considered in the context of impaired white matter growth or more diffuse disturbances of brain maturation rather than only overt destructive lesions [16]. Previous studies have reported associations between ventricular size and impaired neurodevelopment in preterm infants, although the exact parameters and developmental domains have varied across studies [17,18,19]. Therefore, our results support the view that ventricular dimensions at term-equivalent age may serve as a useful indirect marker of neurodevelopment vulnerability.

Head circumference showed positive associations with several developmental domains, both in the early period and at term-equivalent age, although at term-equivalent age statistical significance was observed only for personal skills. These findings are consistent with the broader concept that head growth reflects overall postnatal brain growth. Previous studies have shown associations between poorer postnatal head growth, smaller brain volume, and less favorable neurodevelopmental outcomes in preterm infants [20,21,22]. However, in our cohort, head circumference was less consistently associated with Bayley-4 domains than ventricular width at term-equivalent age, suggesting that it reflects overall postnatal brain growth but should not be interpreted as an independent predictor of neurodevelopmental outcomes. This interpretation is also consistent with previous evidence indicating that head circumference alone has limited sensitivity and specificity for predicting later neurodevelopmental impairment [23].

An additional finding was a positive association between third ventricle width and receptive communication in the early neonatal period, as well as between third ventricle width at term-equivalent age and play and leisure activities. These findings should be interpreted with particular caution. To our knowledge, such specific associations have not been consistently established in the literature, and our study was not designed to explain them mechanistically. They may reflect subtle aspects of maturation of deep midline structures, but they may also represent exploratory findings related to sample size and multiple tested associations [19]. For that reason, these results should be regarded as hypothesis-generating rather than conclusive.

Motor-related outcomes appeared particularly sensitive in this study. This is noteworthy because motor development in preterm infants is known to be closely linked to white matter integrity and overall maturation of periventricular pathways [24]. At the same time, the descriptive analysis also showed relatively broad variability in motor scores, which may have made these domains more likely to reveal associations with morphometric ultrasound parameters.

By contrast, the subarachnoid space, interhemispheric fissure, and Levene index did not show consistent associations with neurodevelopmental outcomes. In this cohort, these parameters appeared less informative than ventricular width [19]. This does not necessarily mean that such measurements are clinically unimportant, but rather that their relationship with early Bayley-4 outcome was less evident in the present exploratory sample.

This study has several limitations. The small sample size limits statistical power and increases the possibility that some observed associations may be unstable. Due to the small sample size, it was not possible to perform multivariable analyses or adjust the results for potential factors that may influence neurodevelopmental outcomes, such as sex, growth restriction, neonatal morbidities, and other clinical characteristics. Another limitation is that interobserver variability was not formally assessed. The exploratory design and absence of adjustment for multiple comparisons also require cautious interpretation. In addition, neurodevelopment was assessed at 12–14 months corrected age, which is still an early developmental time point, and some later-emerging difficulties, especially in higher cognitive and behavioural domains, may not yet have become apparent [25,26]. A separate control group of term-born infants was not included because the primary objective of the study was to evaluate associations within the preterm cohort. Neurodevelopmental outcomes were assessed using standardized Bayley-4 scores referenced to a normative population. Despite these limitations, the study suggests that morphometric cranial ultrasound measurements, particularly ventricular dimensions assessed at term-equivalent age, may provide useful additional information about early neurodevelopment in preterm infants. Head circumference remains an important marker of postnatal growth, but in this cohort, ventricular width at term-equivalent age showed the clearest and most consistent associations with developmental outcomes. These findings support the potential value of combining serial cranial ultrasound morphometry with routine clinical follow-up to identify infants who may require closer developmental surveillance.

## 5. Conclusions

This exploratory study suggests that morphometric cranial ultrasound measurements, particularly those obtained at term-equivalent age, may provide valuable additional information about early brain growth and neurodevelopment in preterm infants. Measurements performed at term-equivalent age demonstrated stronger and more consistent associations with developmental outcomes than those obtained in the early neonatal period, supporting the importance of brain assessment at this developmental stage. Head circumference at term-equivalent age reflects postnatal brain growth but demonstrated less consistent associations with neurodevelopmental outcomes than ventricular morphometric parameters in our cohort, suggesting that it should not be interpreted as a standalone marker of neurodevelopmental outcome. Quantitative ultrasound parameters, particularly ventricular dimensions, may reflect subtle variations in brain maturation that are not detectable through qualitative imaging alone. Our findings suggest that specific cranial ultrasound morphometric parameters are associated with distinct domains of early neurodevelopment. Larger prospective studies with longer follow-up are needed to confirm these findings and define the prognostic value of individual cranial ultrasound morphometric parameters and head circumference for predicting neurodevelopmental outcomes in preterm infants.

## Figures and Tables

**Table 1:** Baseline characteristics of the study population (N = 22).

Characteristic	Value
Male sex, n (%)	9 (40.9)
Female sex, n (%)	13 (59.1)
Gestational age, weeks, mean ± SD	31.1 ± 2.8
Birth weight, g, mean ± SD	1540 ± 620
Birth weight percentile (Fenton 2025 [11]), median (IQR)	47 (25–61)
Birth weight Z-score, mean ± SD	−0.09 ± 1.05
Birth weight < 10th percentile, n (%)	3 (13.6)
Birth weight > 90th percentile, n (%)	1 (4.5)
Birth length, cm, mean ± SD	40 ± 5
Birth length percentile (Fenton 2025 [11]), median (IQR)	37.8 (31.5–51.1)
Head circumference at birth, cm, mean ± SD	28.8 ± 3.0
Head circumference percentile (Fenton 2025 [11]), median (IQR)	55 (28–74)
Head circumference Z-score, mean ± SD	0.12 ± 0.87
Head circumference < 10th percentile, n (%)	1 (4.5)
Head circumference > 90th percentile, n (%)	2 (9.1)
Culture-proven sepsis, n (%)	5 (22.7)
Bronchopulmonary dysplasia, n (%)	1 (4.5)
Retinopathy of prematurity, n (%)	6 (27.3)
Necrotizing enterocolitis, n (%)	2 (9.1)
Intraventricular hemorrhage, n (%)	10 (45.5)

Data are presented as number (n) and percentage (%) for categorical variables and as summary statistics for continuous variables.

## Data Availability

The data presented in this study are available on request from the corresponding author.

## Abbreviations

AGA	Appropriate for gestational age
LVI-R	Levene ventricular index, right side, measured within the first 72 h of life
LVI-L	Levene ventricular index, left side, measured within the first 72 h of life
LVW-R	Lateral ventricle width, right side, measured within the first 72 h of life
LVW-L	Lateral ventricle width, left side, measured within the first 72 h of life
3V	Third ventricle width, measured within the first 72 h of life
SAS	Subarachnoid space width, measured within the first 72 h of life
IHF	Interhemispheric fissure width, measured within the first 72 h of life
HC	Head circumference, measured within the first 72 h of life
LGA	Large for gestational age
LVI-R-TEA	Levene ventricular index, right side at term-equivalent age
LVI-L-TEA	Levene ventricular index, left side at term-equivalent age
LVW-R-TEA	Lateral ventricle width, right side at term-equivalent age
LVW-L-TEA	Lateral ventricle width, left side at term-equivalent age
3V-TEA	Third ventricle width at term-equivalent age
SAS-TEA	Subarachnoid space width at term-equivalent age
IHF-TEA	Interhemispheric fissure width at term-equivalent age
HC-TEA	Head circumference at term-equivalent age
Cog-Scaled	Scaled score, cognitive development
RC-Scaled	Scaled score, receptive communication
EC-Scaled	Scaled score, expressive communication
Lang-Sum	Sum of scaled scores for receptive and expressive communication
FM-Scaled	Scaled score, fine motor scale
GM-Scaled	Scaled score, gross motor scale
Motor-Sum	Sum of scaled scores for fine and gross motor scales
SE-Scaled	Scaled score, socio-emotional scale
SGA	Small for gestational age
AR-Scaled	Scaled score, adaptive receptive behaviour
AE-Scaled	Scaled score, adaptive expressive behaviour
PS-Scaled	Scaled score, personal skills
IR-Scaled	Scaled score, interpersonal relationships
PL-Scaled	Scaled score, play and leisure activities

## References

[1] Song I.G. (2023). Neurodevelopmental outcomes of preterm infants. Clin. Exp. Pediatr..

[2] Rees P., Callanan C., Chadda K.R., Vaal M., Diviney J., Sabti S., Harnden F., Gardiner J., Battersby C., Gale C. (2022). Preterm brain injury and neurodevelopmental outcomes: A meta-analysis. Pediatrics.

[3] Volpe J.J. (2019). Brain injury in premature infants: A complex amalgam of destructive and developmental disturbances. Lancet Neurol..

[4] Back S.A. (2017). White matter injury in the preterm infant: Pathology and mechanisms. Acta Neuropathol..

[5] Inder T.E., Warfield S.K., Wang H., Hüppi P.S., Volpe J.J. (2005). Abnormal cerebral structure is present at term in premature infants. Pediatrics.

[6] de Vries L.S., Cowan F.M. (2009). Evolving understanding of hypoxic-ischemic encephalopathy in the term infant. Semin Pediatr. Neurol..

[7] Toma A.I., Dima V., Rusu L., Nemeș A.F., Gonț B.F., Arghirescu A., Necula A., Fieraru A., Stoiciu R., Andrășoaie L. (2025). Cerebral ultrasound at term-equivalent age: Correlations with neuro-motor outcomes at 12–24 months corrected age. Children.

[8] Bayley N., Aylward G.P. (2019). Bayley Scales of Infant and Toddler Development.

[9] Anderson P.J., Burnett A. (2017). Assessing developmental delay in early childhood: Concerns with the Bayley-III scales. Clin. Neuropsychol..

[10] R Core Team (2022). R: A Language and Environment for Statistical Computing.

[11] Fenton T.R., Elmrayed S., Alshaikh B.N. (2025). Fenton Third-Generation Growth Charts of Preterm Infants Without Abnormal Fetal Growth: A Systematic Review and Meta-Analysis. Paediatr. Perinat. Epidemiol..

[12] Back S.A., Miller S.P. (2014). Brain injury in premature neonates: A primary cerebral dysmaturation disorder?. Ann. Neurol..

[13] Hintz S.R., O’Shea M. (2008). Neuroimaging and neurodevelopmental outcomes in preterm infants. Semin. Perinatol..

[14] Kidokoro H., Neil J.J., Inder T.E. (2013). New MR imaging assessment tool to define brain abnormalities in very preterm infants at term. AJNR Am. J. Neuroradiol..

[15] Brouwer M.J., van Kooij B.J., van Haastert I.C., Koopman-Esseboom C., Groenendaal F., de Vries L.S., Benders M.J. (2014). Sequential cranial ultrasound and cerebellar diffusion weighted imaging contribute to the early prognosis of neurodevelopmental outcome in preterm infants. PLoS ONE.

[16] Brouwer M.J., de Vries L.S., Groenendaal F., Koopman C., Pistorius L.R., Mulder E.J., Benders M.J. (2012). New reference values for the neonatal cerebral ventricles. Radiology.

[17] Reis J.D., Hagan T., Heyne R., Tolentino-Plata K., Clarke R., Brown L.S., Rosenfeld C.R., Burchfield P.J., Caraig M., Brion L.P. (2024). Relationship between ventricular size on latest ultrasonogram and the bayley scores ≥ 18 months in extremely low gestational age neonates: A retrospective cohort study. Am. J. Perinatol..

[18] Sheng M., Guo T., Mabbott C., Chau V., Synnes A., de Vries L.S., Grunau R.E., Miller S.P. (2022). Ventricular volume in infants born very preterm: Relationship with brain maturation and neurodevelopment at age 4.5 years. J. Pediatr..

[19] Franckx H., Hasaerts D., Huysentruyt K., Cools F. (2018). Cranial ultrasound and neurophysiological testing to predict neurological outcome in infants born very preterm. Dev. Med. Child Neurol..

[20] Raghuram K., Yang J., Church P.T., Cieslak Z., Synnes A., Mukerji A., Shah P.S., for the Canadian Neonatal Network, Canadian Neonatal Follow-Up Network Investigators (2017). Head growth trajectory and neurodevelopmental outcomes in preterm neonates. Pediatrics.

[21] Selvanathan T., Guo T., Kwan E., Chau V., Brant R., Synnes A.R., E Grunau R., Miller S.P. (2022). Head circumference, total cerebral volume and neurodevelopment in preterm neonates. Arch. Dis. Child. Fetal Neonatal Ed..

[22] Neubauer V., Griesmaier E., Pehböck-Walser N., Pupp-Peglow U., Kiechl-Kohlendorfer U. (2013). Poor postnatal head growth in very preterm infants is associated with impaired neurodevelopmental outcome. Acta Paediatr..

[23] Wright C.M., Emond A. (2015). Head growth and neurocognitive outcomes. Pediatrics.

[24] Spittle A.J., Cameron K., Doyle L.W., Cheong J.L., Victorian Infant Collaborative Study Group (2018). Motor Impairment Trends in Extremely Preterm Children: 1991–2005. Pediatrics.

[25] Anderson P.J., De Luca C.R., Hutchinson E., Roberts G., Doyle L.W., Victorian Infant Collaborative Group (2010). Underestimation of developmental delay by the new Bayley-III Scale. Arch. Pediatr. Adolesc. Med..

[26] Kipping S.M., Kiess W., Ludwig J., Meigen C., Poulain T. (2024). Are the Results of the Bayley Scales of Infant and Toddler Development (Third Edition) Predictive for Later Motor Skills and School Performance?. Children.

